# Most deaths in low-risk cardiac surgery could be avoidable

**DOI:** 10.1038/s41598-020-80175-7

**Published:** 2021-01-13

**Authors:** Omar Asdrúbal Vilca Mejia, Gabrielle Barbosa Borgomoni, Eduardo Gomes Lima, Gustavo Pampolha Guerreiro, Luís Roberto Dallan, Pedro de Barros e Silva, Marcelo Arruda Nakazone, Orlando Petrucci Junior, Walter José Gomes, Marco Antonio Praça de Oliveira, Alexandre Sousa, Valquíria Pelisser Campagnucci, Marcos Gradim Tiveron, Alfredo José Rodrigues, Rafael Ângelo Tineli, Roberto Rocha e Silva, Luiz Augusto Ferreira Lisboa, Fabio Biscegli Jatene

**Affiliations:** 1 Department of Cardiovascular Surgery, Universidade de São Paulo Instituto do Coração (INCOR), São Paulo, São Paulo Brazil; 2 Department of Cardiovascular Surgery, Hospital Samaritano Paulista, São Paulo, São Paulo Brazil; 3 Department of Cardiovascular Surgery, Hospital De Base de São José do Rio Preto, São José de Rio Preto, São Paulo Brazil; 4 Department of Cardiovascular Surgery, Universidade Estadual de Campinas (UNICAMP), Campinas, São Paulo Brazil; 5 Department of Cardiovascular Surgery, Universidade Federal de São Paulo (UNIFESP), São Paulo, São Paulo Brazil; 6 Department of Cardiovascular Surgery, Beneficência Portuguesa de São Paulo, São Paulo, São Paulo Brazil; 7 Department of Cardiovascular Surgery, Irmandade da Santa Casa de Misericórdia de São Paulo, São Paulo, São Paulo Brazil; 8 Department of Cardiovascular Surgery, Irmandade da Santa Casa de Misericórdia de Marília, Marília, São Paulo Brazil; 9 Departament of Cardiovascular Surgery, Universidade de São Paulo Hospital das Clínicas da Faculdade de Medicina de Ribeirão Preto, São Paulo, Brazil; 10 Department of Cardiovascular Surgery, Irmandade da Santa Casa de Misericórdia de Piracicaba, Piracicaba, São Paulo Brazil; 11 Department of Cardiovascular Surgery, Hospital Paulo Sacramento, Jundiaí, São Paulo Brazil

**Keywords:** Cardiology, Interventional cardiology, Risk factors

## Abstract

It is observed that death rates in cardiac surgery has decreased, however, root causes that behave like triggers of potentially avoidable deaths (AD), especially in low-risk patients (less bias) are often unknown and underexplored, Phase of Care Mortality Analysis (POCMA) can be a valuable tool to identify seminal events (SE), providing valuable information where it is possible to make improvements in the quality and safety of future procedures. Our results show that in São Paul State, only one third of AD in low-risk cardiac surgery was related to specific surgical problems. After a revisited analysis, 75% of deaths could have been avoided, which in the pre-operative phase, the SE was related judgment, patient evaluation and preparation. In the intra-operative phase, most occurrences could have been avoided if other surgical technique had been used. Sepsis was responsible for 75% of AD in the intensive care unit. In the ward phase, the recognition/management of clinical decompensations and sepsis were the contributing factors. Logistic regression model identified age, previous coronary stent implantation, coronary artery bypass grafting + heart valve surgery, ≥ 2 combined heart valve surgery and hospital-acquired infection as independent predictors of AD.

## Introduction

Mortality rates due to cardiac surgery have been decreasing across the world^1–3^, however, avoidable deaths still occur in low-risk patients^4,5^. Traditional analysis methods of cardiac surgeries outcomes focus on adjusting mortality rates, without objectively analyzing the root causes of problems; therefore, nonsurgical factors lose significance and are often unknown and underexplored^4^.

In 2012, a team from the Michigan Society of Thoracic and Cardiovascular Surgeons (MSTCVS) developed a new structured approach for the review of mortality called Phases of Care Mortality Analysis (POCMA)^6^. For this study, POCMA focuses on low-risk patients analyzing patients with a lower probability of negative results, allowing to identify objectively where the avoidable error occurred. For the analysis, a patient with a lower risk of complications has less bias in the analysis. Therefore, the application of POCMA in this subgroup can generate one of the best learning opportunities for quality care in patients undergoing cardiac surgery.

In aviation, the cause of an accident begins with a seminal event that is always associated with other contributing factors; thus, a cascade of factors is aligned in a random process that influences the outcome. A system as complex and dynamic as civil aviation resembles the health system because its outcomes can be affected by multiple sequential factors^5,7–9^. To identify the root cause of death in cardiac surgery, objective methods are essential to enhance patient safety and improve results. In this aspect, cardiac surgeons have been pioneers in the systematic analysis of data related to continuous improvement of outcomes^10,11^.

High-risk patients are more likely to have a poor outcome because of their comorbidities and confounding factors^12^; therefore, identifying problems that happen to low-risk patients would help to better understand our practice^13^. In a next level, improvements will be brought to all patients undergoing cardiac surgery^14^. This study aims to assess, through POCMA, the triggers of potentially avoidable surgical deaths of low-risk patients.

## Methods

### Population and sample

The analysis was based on a multicenter, mandatory registry that includes data collected between November 2013 and January 2016 performed in 10 hospitals in São Paulo State. Data collection and follow-up were carried out by trained people (approved, and continuously monitored for this objective in each participating center). Data were incorporated into the website http://bdcardio.incor.usp.br/, through 4 available interfaces, including pre-operative, intra-operative, post-operative and 30-day evaluation. The patient follow-up was carried out through telephone interviews. Data integrity and veracity were overseen by the executive records committee. Variable definitions were adopted from the European System for Cardiac Operative Risk Evaluation II (EuroSCORE II), in which 68 variables are collected per patient and the mortality risk was calculated at http://www.EuroSCORE.org/calc.html. All EuroSCORE values of these patients were recalculated and validated by the POCMA multidisciplinary team.

Patients with 18 years or older and EuroSCORE II ≤ 2 were included in this analysis, regardless of being elective, urgent or emergency cardiac surgery. It is worth mentioning that patients with EuroSCORE II > 2, but who were initially underestimated, were also included in this analysis and considered as errors in the pre-operative evaluation.

### Data analysis and outcomes

The POCMA analysis was performed by a multidisciplinary and technical group composed of cardiologist, cardiac surgeon, intensive care physician, nurse and a perfusionist from the coordinating center. Using POCMA, the identified seminal event (SE) was then categorized according to the phase of the event during peri-operative care (pre-operative, intra-operative, post-operative intensive care unit [ICU] phase and post-operative floor [ward] phase). A list of categories and subcategories capable of triggering mortality was developed based on POCMA for each perioperative phase^6^. When multiple contributing factors were found, the first potential event was chosen, representing the best time for systematic correction of the course of mortality. Death of cardiac surgery patients was defined as avoidable if the chance of survival with better care or in the absence of contributing factors was > 50%. When there were no identifiable factors for a sudden death of a patient, it was defined as unavoidable death (catastrophic event).

### Statistical analysis

In the descriptive analysis, continuous variables were expressed in terms of summary measures (mean, median, standard deviation, and quartiles), while categorical variables were expressed in terms of percentages. To compare two groups of continuous variables, the t-test was used when they followed a normal distribution (Anderson–Darling test). For the others, Mann–Whitney and Brunner–Munzel non-parametric tests were used, respectively, for homogeneous and heterogeneous variables (Bartlett test). For categorical variables, Fisher's exact test or the chi-square test was used. To find associations between the explanatory variables and the outcome, the logistic regression model was used. To evaluate the multiple model, the Hosmer–Lemeshow test was performed and the C statistic (C statistics) was calculated. The level of significance adopted in the tests was 0.05. Two-tailed hypotheses were considered. The R statistical software version 3.6.0 was used to perform all analyses ^15^.

The Inter-rater Reliability method was used to evaluate the reliability in the process of identifying the seminal event using the POCMA tool and classifying deaths as avoidable or unavoidable. The percentage of agreement among the multidisciplinary team was 79.7% for avoidable deaths and 89.5% for unavoidable deaths (Supplementary file 1).

### Ethics and consent

This study is part of the project: “Heart surgery programs innovation using surgical risk stratification at the São Paulo State Public Healthcare System: SP-SCORE-SUS STUDY” approved with the number 3853/12/109 by the Ethics Committee of the Heart Institute of the Hospital das Clínicas, Medicine School, University of São Paulo, Brazil. http://www.incor.usp.br/sites/incor2013/index.php/equipe/16-pesquisa/comissao-cientifica/158-fale-conosco. The free and informed consent was waived due to the analysis of pre-established data logs. We declare that all methods were performed in accordance with relevant guidelines and regulations.

## Results

**Figure 1:**
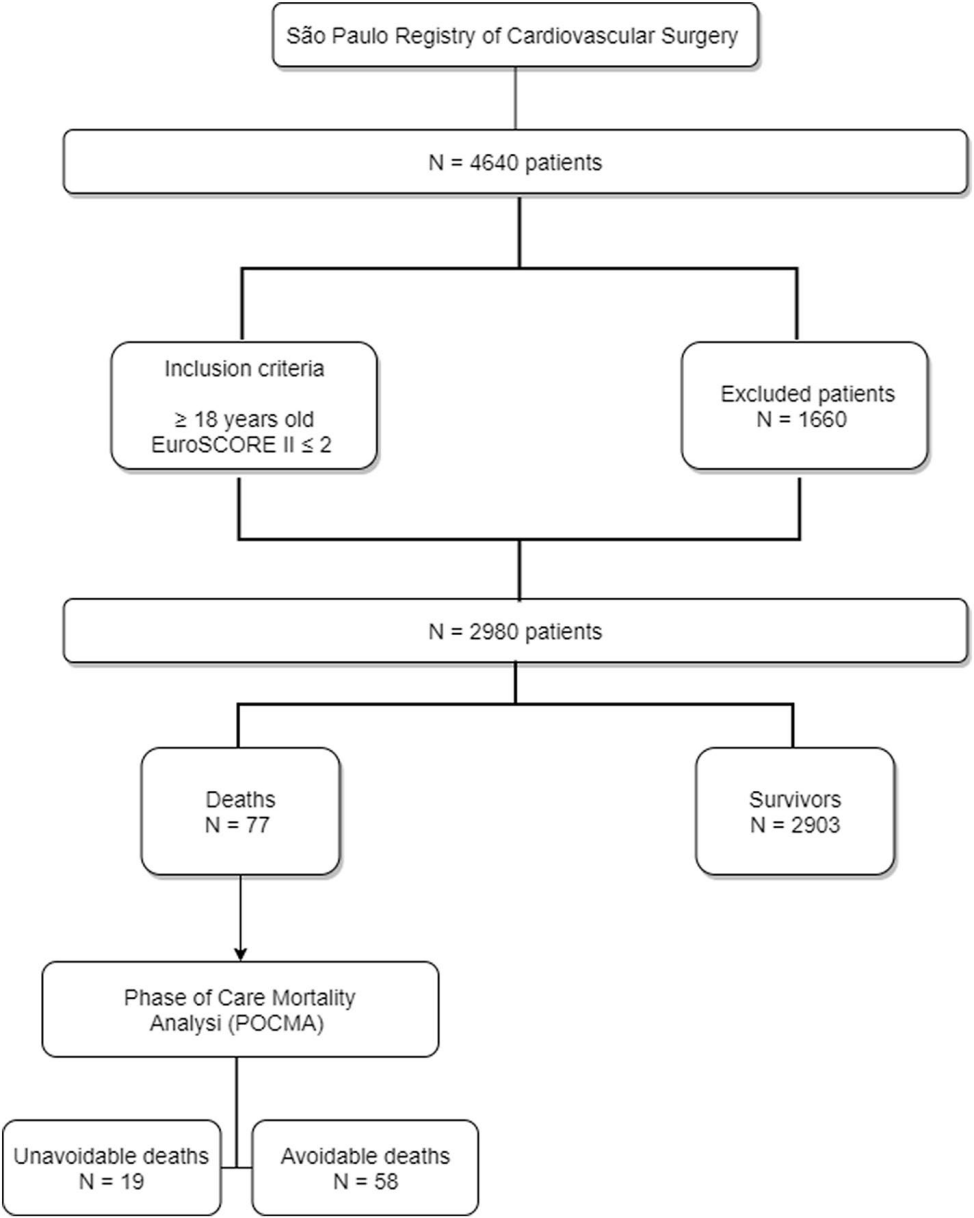
Flowchart of patient selection in the São Paulo Phase of Care Mortality Analysis study. Description about the selection of patients used for this analysis.

All patient data were obtained from the São Paulo Registry of Cardiovascular Surgery and stratified by EuroSCORE II. In total, 4640 patients underwent cardiac surgery during the study period. Among these, 2980 were preoperatively considered low-risk patients (EuroSCORE II ≤ 2), 77 patients died (2.6%), 58 patients were deemed avoidable deaths (75%) and 19 patients were considered unavoidable deaths (25%) by the multidisciplinary POCMA team, as described in Fig. 1.

As there were 58 patients in the “Avoidable deaths” group and 2903 in the “Survivors” group, there could be a bias to perform comparative analyses. Therefore, a random sample of 232 patients from the Survivors group (proportion of 20%/80%) was performed. Of importance, the surviving patients were younger (58.4 vs. 61.5 years old, *p* = 0.049), the majority were male (75% vs. 62%, *p* = 0.048), had higher body mass index (28.6 vs. 25.6, *p* =  < 0.001) and less than half had previous coronary stent (8% vs. 19%, *p* = 0.027). In general, the surviving patients had more isolated coronary artery bypass grafting (CABG) procedures (*p* = 0.004) and had a lower risk as stratified by STS and EuroSCORE II (*p* < 0.001). The only factor significantly worse in the surviving patients was higher glycated hemoglobin (6.9% vs. 6.3%, *p* = 0.026). Supplementary file 2 provides clinical data for both groups (surviving and avoidable death patients) in our database.

In the avoidable deaths group, the New York Heart Association (NYHA) functional classification was 22.4% in class I, 39.7% in class II, 32.8% in class III and 5.2% in class IV. The types of surgical procedures were isolated CABG (50%), CABG+ heart valve surgery (10.4%), isolated valve surgery (mitral, aortic or tricuspid valve surgery, 29.3%), combined valves surgeries (≥ 2 valves, 8.6%) and other type of combined procedures (1.7%). In the current group, 72.4% were hospitalized for elective surgery and 27.6% for urgent procedures.

**Figure 2:**
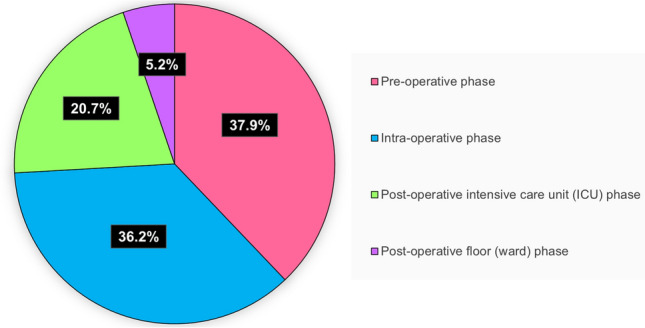
Phase of care mortality analysis seminal event in hospitalization phases. Using the POCMA analysis tool, the SE were categorized according to the perioperative phase of care.

**Figure 3:**
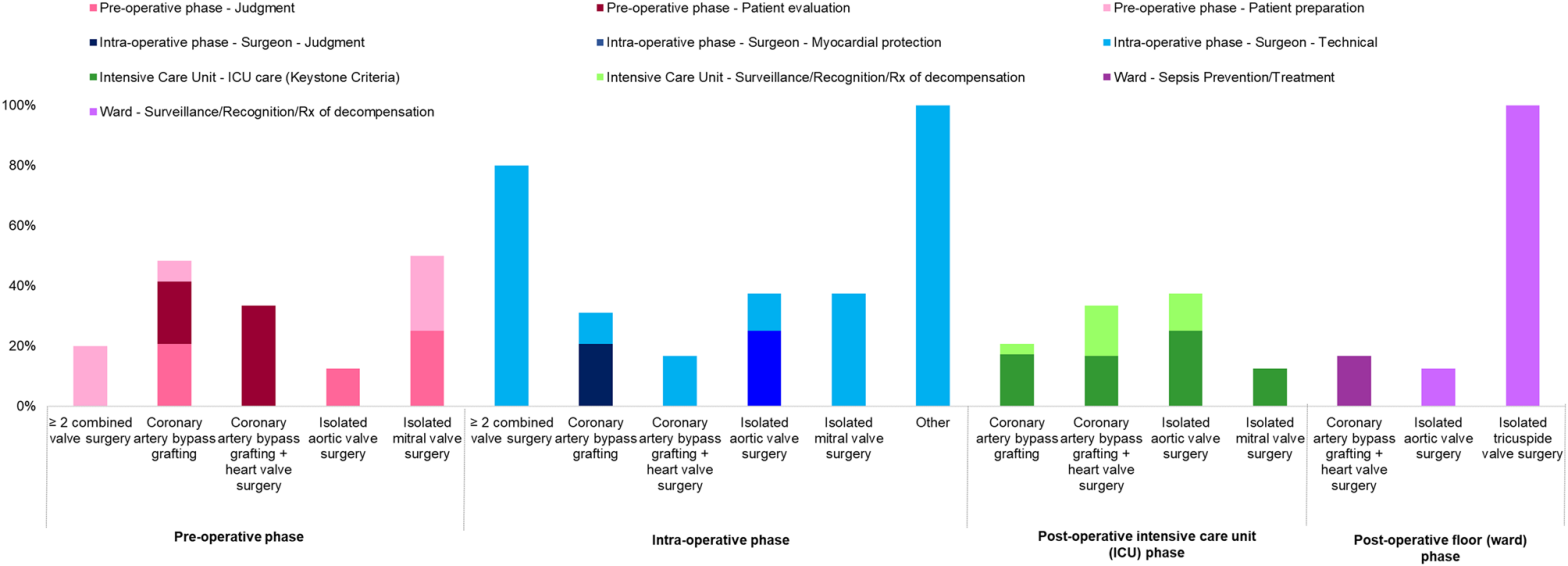
Phase of care mortality analysis comparison between surgery populations. Association between perioperative care phase SE and surgical procedure.

Using the POCMA tool, the identified SE was then categorized according to the phase of the event during the perioperative care^6^. In 22 (37.9%) of the avoidable deaths, SE was originated in the pre-operative phase, 21 (36.2%) in the intra-operative phase, 12 (20.7%) in the intensive care unit, and 3 (5.2%) in the ward phase (Fig. 2). In Fig. 3 and in Supplementary file 3, the incidence of SE related to the patient’s care phase in the perioperative period, the association between SE and the type of surgical procedure, as well as the events that led to their death were described. It was observed that, of the 58 avoidable deaths, 38 (65.5%) patients died from heart disease (cardiogenic shock), 15 (25.9%) from septic shock, 2 (3.4%) from multiple organ failure, 1 (1.7%) from hypovolemic shock and 1 (1.7%) from vasoplegic shock.

On the other hand, the 19 unavoidable deaths, considered catastrophic events by the multidisciplinary POCMA team, are described in the Supplementary file 4. It was observed that 3 (15.8%) patients died from severe and refractory vasoplegic syndrome, 2 (10.5%) from severe and refractory arrhythmias and 1 each (5.3%) from severe and refractory acute respiratory failure, cefepime-induced encephalopathy, severe and refractory systemic inflammatory response syndrome (SIRS), major impairment of brain self-regulation, severe and refractory pulmonary hypertension, cardiac arrest with pulseless electrical activity, perforation of the abdominal aorta after passage of an intra-aortic balloon, sepsis, ventricular fibrillation and cardiac arrest, severe and refractory status epilepticus, acute mesenteric ischemia, iatrogenic aortic dissection, severe and refractory bleeding and from severe and refractory postoperative clinical decompensation.

During the pre-operative phase (37.9%), SE were related to: (1) inadequate judgment of surgical indications (15.5%), divided into two subcategories, whereas the timing of surgery was responsible for 1.7% of these cases and risk > benefit 13.8%; (2) patient evaluation, with inadequate risk identification (13.8%) and; (3) patient preparation without medical status optimization (8.6%). Errors in the EuroSCORE pre-operative calculation led to a failure to identify some risk factors that could have led to optimized strategies or even refusal of treatment at the time of indication. For example, a case in which a patient with chronic kidney disease (creatinine clearance = 82 ml/min) was not identified by the participating center and, therefore, was not included in the EuroSCORE II calculation. Therefore, this patient was not optimized for planning and preparing before, during and after surgery.

In the intra-operative phase (36.2%), the SE were surgeon-related issues as intra-operative technical errors (22.4%), inadequate surgical judgment (10.4%) and problems with myocardial protection (3.4%). In some cases, the surgeon defined to perform the surgery without the use of cardiopulmonary bypass (CPB), but hemodynamic instability needed CPB to finish the procedure. Approximately 32.8% of occurrences would potentially be avoided if a different strategy or surgical technique was used.

During the intensive care unit phase, which included 20.7% of the total SE in our analysis, the subcategories were about sepsis prevention/treatment (15.5%) and surveillance/recognition/treatment of decompensations (5.2%).

As for the ward phase (5.2%), problems with sepsis prevention/treatment (1.7%), as well as surveillance/recognition/treatment of other decompensations (3.5%) were related to deaths.

Thus, the deaths occurred due to the failure in the early identification of the problem for the respective aggressive approach. In summary, among deaths occurring in patients considered to be at low risk for cardiac surgery, 75% would be avoidable deaths according the multidisciplinary POCMA team.

A regression analysis was performed, with the outcome variable being "death" within our database selected as low-risk patients by EuroSCORE II (≤ 2), excluding the 19 catastrophic events. Using the variables that were significant in the simple regressions, the multiple model was built (Table 1).

**Table 1:** Multiple regression model. CI confidence interval; * p value < 0.05.

Explanatory variable	Coefficient	Standard error coefficient	OR	CI95%	*p* value
Age	0.04	0.02	1.04	1.01–1.08	0.014*
Previous coronary stent	1.40	0.46	4.07	1.65–10.8	0.002*
Coronary artery bypass grafting + heart valve surgery	1.76	0.65	5.80	1.61–20.86	0.007*
≥ 2 combined heart valve surgery	1.91	0.70	6.78	1.72–26.76	0.006*
Hospital-acquired infection	0.95	0.39	2.59	1.21–5.54	0.014*

**Figure 4:**
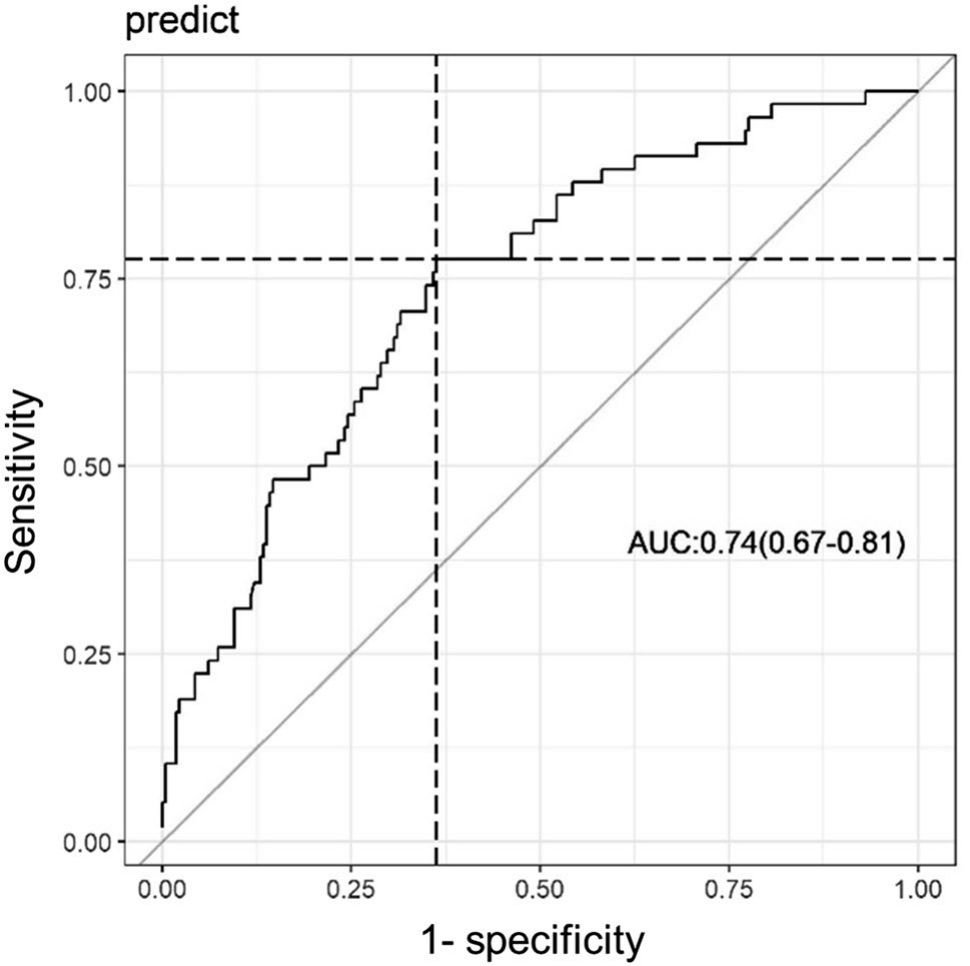
ROC curve of the São Paulo Phase of Care Mortality Analysis study. Figure was generated using R statistical software version 3.6.0^15^.

To test the performance of the model to predict mortality, the Hosmer–Lemeshow tests (with g = 10) and C statistics (C statistics) were calculated. The Hosmer–Lemeshow test (*p* = 0.3709) showed that the model was well calibrated. Moreover, C statistics (0.74, 95% CI 0.67–0.81) revealed that the multiple model is accurate to predict death in low-risk cardiac surgeries (Fig. 4).

## Discussion

Cardiac surgery achieves excellent results, even in complex procedures or in high-risk patients. However, classic morbidity and mortality assessment does not evaluate the impact of multidisciplinary care on the systematic approach^12,16^. In the aviation industry, evaluate SE and contributing factors to poor outcomes is important for a cycle of continuous quality improvement^17^. Thus, the emergence of POCMA for perioperative mortality assessment in cardiac surgery has allowed the introduction of a rational and objective method^6^.

Freed et al., in 2009, reported the mortality rate from cardiac surgery in patients with logistic EuroSCORE ≤ 2 (CABG and heart valve surgery) in a Cambridge hospital as an infrequent event (0.37%)^13^. More recently, in 2013, a study conducted in Istanbul, by Cakalagaoglu et al., showed 0.93% of deaths from CABG the low-risk group (EuroSCORE ≤ 2)^18^.

In our study, mortality after isolated CABG was 1.9%, different than the rate of 0.5% found by Rubino et al.^19^ The main reason for this divergence may be that, in our study, we include patients at higher risk (EuroSCORE II = 2 and > 2 due to cases with miscalculation in the pre-operative phase), acute coronary syndromes, left ventricular ejection fraction < 30%, critical pre-operative status, non-elective surgery, age > 80 years, poor mobility, prior cardiac surgery and chronic kidney disease. This would explain why we use 64% versus 32% of the total cardiac surgery population.

Our study included all types of cardiac procedures, as well as a Sweden study, however our percentage of avoidable mortality was higher (75% vs. 55%)^5^. There are some important differences in both analyses: our study was multicentric, used a single patient selection risk score (EuroSCORE II) and the analysis of deaths was performed by an independent multidisciplinary POCMA team.

In our study, 36.2% of avoidable deaths occurred during the intra-operative phase. Similar results were found in 1558 cardiac surgery patients in the United Kingdom, where 37.3% of deaths were related to intra-operative factors, including patients from all risk groups. It confirms that only one third of deaths after cardiac surgery could be avoided in the operating theatre. In the present study, 45 patients with avoidable deaths (77.6% of patients who died) had non-technical errors in the intra-operative phase, similar to what was observed in the United Kingdom after performing autopsies (86%)^11^. This means that most avoidable deaths cannot be related to errors in the surgical technique, regardless of the type of cardiac surgery and the surgical risk profile.

The same study found that complications in high-risk patients were usually more due to the environment outside the operating theatre^11^. Our results showed that the failure process frequently occurs in the pre-operative phase of cardiac surgery, confirming that this occurs regardless of the patient’s risk.

Guru et al. found an avoidable mortality rate of 32% for isolated CABG^4^. According to the analysis by Shannon et al., 41% of all deaths were avoidable, as well as 42% of deaths from isolated CABG^6^. We found that 75% of deaths could be avoided, perhaps because we only analysed low-risk patients. Our percentage of avoidable death is similar to that found by Rubino et al., although they used only an isolated CABG sample^19^.

Since 2008, Guru et al. have warned of the need to access and audit unfavorable outcomes for improvement in the process^4^. Their analysis also found that inadequate surgical judgment was responsible for 46% of intra-operative problems. This showed that planning changes when already in the operating theatre, would be completely attributed to the surgeon, whereas in fact a multidisciplinary perioperative evaluation demonstrated a significant difference for outcomes in cardiac surgery patients. When analyzed in a single institution, these can be considered isolated incidents, but when viewed from a multicentric perspective, opportunities for quality improvement becomes clearer^13^.

A new aspect of this analysis is that we included the miscalculation in the risk score (EuroSCORE II) as pre-operative error (risk identification). The error was observed in 13.8% of patients in this study. The initial appraisal was considered by the current analysis because patients were referred for surgery with this risk prediction, confirming errors in surgical planning.

The independent predictors of mortality in this present population of low-risk cardiac surgery patients were: age, previous coronary stent, CABG + heart valve surgery, ≥ 2 combined heart valve surgery and hospital-acquired infection. Our findings are supported by the scientific literature^20–23^. These results confirm that patients with previous coronary stent, even if considered of low risk, have worse results after cardiac surgery. On the other hand, there are high-risk procedures that even in low-risk patients are associated with increased mortality rates.

Undoubtedly, results will improve over time, with continuous quality improvement policies focused on discussions about the risk–benefit of surgery, surgery timing, and the adoption of strategies from highly reliable organizations^5,13,14,24–26^, since there are no improvements without identifying problems and measuring the results of surgical procedures^27^. As such, all discussions, especially for more complex cases, would result in a more balanced appraisal of the role of cardiac surgery in patients with a limited life expectancy. Thus, reducing judgment errors during the pre-operative phase of high-risk patients would reflect a systematic improvement, giving more "awareness" to the surgical indication in this phase, reassuring the importance of the Heart Team as a quality improvement measure^11^.

This analysis involved only low-risk patients according to EuroSCORE II, with the intention of having a more homogeneous sample, therefore, a greater probability of detecting avoidable deaths. This resulted in a sample with less complex patients, in which systematic errors in perioperative care were more exposed. Thus, it identified learning opportunities through POCMA, such as failure in patient preparation and objective evaluation, failure in surgical judgment, failure to identify higher-risk cases, and changes in planning in the operating theatre, leading to an increase in surgery time and, consequently, in avoidable deaths.

Preliminary analyses such as these are the first steps in changing how to deal with a worldwide “culture of error”. Several studies illustrate that identifying failures contributes to eradicate them from complex processes such as civil aviation and cardiac surgery^17,28^. The “Death in low-risk cardiac surgery: the failure to achieve a satisfactory cardiac outcome (FIASCO) study” showed a clear decrease in systematic errors after identification and correction of the main contributing factors related to the death of low-risk patients, confirming the relevance of our study^13^.

This analysis has intrinsic limitations: (1) it is a retrospective analysis, but in a prospective registry. Potential criticism can come from the reliability of the main surgeon or the institution itself to properly judge their own mistakes. Therefore, a multidisciplinary group was responsible for the assessment of deaths; (2) the autopsy data could have helped to close the cause of death. However, these are different analyses, POCMA focuses on identifying the perioperative phase in which the SE (failure in care) occurred, however, the autopsy only analyses the cause of death.

In our study, we describe the evolution of a quality initiative in a state-level approach, emphasizing an analytical tool (POCMA) that uses a multidisciplinary review method to evaluate SE of avoidable deaths^8^. Patients referred for cardiac surgery should be properly stratified, and the treatment should always be discussed in a multidisciplinary approach, as the execution of a high-risk procedure, even in clinically low-risk patients, needs to be planned and surgically monitored through well-established and double-checked protocols^13^.

It must be emphasized that the SE identified were stratified by phase and not by the professional involved in the process, reinforcing the importance of the multidisciplinary team in assessing all cases by a systematic approach^11,13^.

## Conclusion

Only one third of deaths in low-risk cardiac surgery were related to specific surgical problems. Most seminal events were allocated outside the surgical environment. Finally, 75% of the total low-risk deaths could be avoidable. Multicentric and mandatory registries generate real-world data, where opportunities for improving the safety of surgical patients can be identified through multidisciplinary and systematic analyses.

## Supplementary information

Supplementary information 1.

Supplementary information 2.

Supplementary information 3.

Supplementary information 4.
