# Supplementary material for: Most deaths in low-risk cardiac surgery could be avoidable
Source: Sci Rep. 2021 Jan 13;11:1045. doi: 10.1038/s41598-020-80175-7 (PMC7806717; doi:10.1038/s41598-020-80175-7)
Supplement: Supplementary file 1 — Supplementary information 1. [file 41598_2020_80175_MOESM1_ESM.pdf]

Most deaths in low-risk cardiac surgery could be avoidable

Omar Asdrúbal Vilca Mejia[1,2,*], Gabrielle Barbosa Borgomoni[1], Eduardo Gomes Lima[1, +], Gustavo Pampolha Guerreiro[1,+], Luís Roberto Palma Dallan[1], Pedro Gabriel Melo de Barros e Silva[2,+], Marcelo Arruda Nakazone[3,+], Orlando Petrucci Junior[4,+], Walter José Gomes[5,+], Marco Antonio Praça de Oliveira[6,+], Alexandre Sousa[6,+], Valquíria Pelisser Campagnucci[7,+], Marcos Gradim Tiveron[8,+], Alfredo José Rodrigues[9,+] Rafael Ângelo Tineli[10,+], Roberto Rocha e Silva[11,+], Luiz Augusto Ferreira Lisboa[1], Fabio Biscegli Jatene[1].

[1] Department of Cardiovascular Surgery, Universidade de São Paulo Instituto do Coração (INCOR), São Paulo, São Paulo, Brazil.

[2] Department of Cardiovascular Surgery, Hospital Samaritano Paulista, São Paulo, São Paulo, Brazil.

[3] Department of Cardiovascular Surgery, Hospital De Base de São José do Rio Preto, São José de Rio Preto, São Paulo, Brazil.

[4] Department of Cardiovascular Surgery, Universidade Estadual de Campinas (UNICAMP), Campinas, São Paulo, Brazil.

[5] Department of Cardiovascular Surgery, Universidade Federal de São Paulo (UNIFESP), São Paulo, São Paulo, Brazil.

[6] Department of Cardiovascular Surgery, Beneficência Portuguesa de São Paulo, São Paulo, São Paulo, Brazil.

[7] Department of Cardiovascular Surgery, Irmandade da Santa Casa de Misericórdia de São Paulo, São Paulo, São Paulo, Brazil.

[8] Department of Cardiovascular Surgery, Irmandade da Santa Casa de Misericórdia de Marília, Marília, São Paulo, Brazil.

[9] Departament of Cardiovascular Surgery, Universidade de São Paulo Hospital das Clínicas da Faculdade de Medicina de Ribeirão Preto, São Paulo, Brazil.

[10] Department of Cardiovascular Surgery, Irmandade da Santa Casa de Misericórdia de Piracicaba, Piracicaba, São Paulo, Brazil.

[11] Department of Cardiovascular Surgery, Hospital Paulo Sacramento, Jundiaí, São Paulo, Brazil.

*Corresponding author: E-mail: omar.mejia@incor.usp.br.

+these authors contributed equally to this work.

Supplementary file 1

POCMA agreement among the multiprofessional analysis

Avoidable Deaths

Cardiac surgeon	Perfusionist	Intensive care physician	Cardiologist	Nurse	Agreement POCMA result N
	x	x	x	x	4
x	x	x	x	x	5
x		x	x	x	4
x	x	x	x	x	5
x	x		x	x	4
	x	x	x	x	4
x	x	x	x		4
x	x	x	x	x	5
x	x	x	x	x	5
x	x	x	x	x	5
x	x	x		x	4
x	x	x		x	4
x	x	x	x	x	5
x	x	x	x	x	5
	x	x	x	x	4
x	x	x	x	x	5
x	x	x	x	x	5
x	x	x	x	x	5
x	x	x		x	4
x	x	x	x	x	5
x	x	x		x	4
x	x	x	x	x	5
x	x	x	x	x	5
x	x	x	x	x	5
x	x	x	x	x	5
x	x	x	x	x	5
	x	x	x	x	4
x	x	x	x	x	5
x	x	x	x	x	5
x	x	x		x	4
	x	x	x	x	4
x	x	x	x	x	5
x	x	x	x	x	5
x	x		x		3
x	x	x	x	x	5
x	x		x	x	4
x	x	x		x	4
x	x	x	x	x	5
x	x	x		x	4
		x	x	x	3
x	x	x	x	x	5
x	x	x	x	x	5
x	x	x	x	x	5
x	x			x	3
x	x		x	x	4
x	x	x		x	4
x	x	x		x	4
x	x	x	x	x	5
	x	x	x	x	4
x	x		x	x	4
	x	x	x	x	4
	x	x	x	x	4
x	x	x	x	x	5
x	x	x	x	x	5
	x	x	x	x	4
x	x	x	x	x	5
x	x		x	x	4
x	x	x	x	x	5

Unavoidable deaths

Cardiac surgeon	Perfusionist	Intensive care physician	Cardiologist	Nurse	Agreement POCMA result N
	x	x	x	x	4
x	x	x	x	x	5
x	x	x	x	x	5
	x	x	x	x	4
x	x	x	x	x	5
x	x	x	x	x	5
x	x		x	x	4
x	x	x	x	x	5
x	x	x	x	x	5
x	x	x	x	x	5
x	x		x	x	4
x	x	x	x	x	5
x	x	x	x	x	5
x	x	x	x	x	5
x	x	x	x	x	5
x	x	x	x	x	5
x	x	x	x	x	5
x	x	x	x	x	5
x	x		x	x	4

x : agreement.
