# Supplementary material for: Most deaths in low-risk cardiac surgery could be avoidable
Source: Sci Rep. 2021 Jan 13;11:1045. doi: 10.1038/s41598-020-80175-7 (PMC7806717; doi:10.1038/s41598-020-80175-7)
Supplement: Supplementary file 2 — Supplementary information 2. [file 41598_2020_80175_MOESM2_ESM.pdf]

Most deaths in low-risk cardiac surgery could be avoidable

Omar Asdrúbal Vilca Mejia[1,2,*], Gabrielle Barbosa Borgomoni[1], Eduardo Gomes Lima[1, +], Gustavo Pampolha Guerreiro[1,+], Luís Roberto Palma Dallan[1], Pedro Gabriel Melo de Barros e Silva[2,+], Marcelo Arruda Nakazone[3,+], Orlando Petrucci Junior[4,+], Walter José Gomes[5,+], Marco Antonio Praça de Oliveira[6,+], Alexandre Sousa[6,+], Valquíria Pelisser Campagnucci[7,+], Marcos Gradim Tiveron[8,+], Alfredo José Rodrigues[9,+] Rafael Ângelo Tineli[10,+], Roberto Rocha e Silva[11,+], Luiz Augusto Ferreira Lisboa[1], Fabio Biscegli Jatene[1].

[1] Department of Cardiovascular Surgery, Universidade de São Paulo Instituto do Coração (INCOR), São Paulo, São Paulo, Brazil.

[2] Department of Cardiovascular Surgery, Hospital Samaritano Paulista, São Paulo, São Paulo, Brazil.

[3] Department of Cardiovascular Surgery, Hospital De Base de São José do Rio Preto, São José de Rio Preto, São Paulo, Brazil.

[4] Department of Cardiovascular Surgery, Universidade Estadual de Campinas (UNICAMP), Campinas, São Paulo, Brazil.

[5] Department of Cardiovascular Surgery, Universidade Federal de São Paulo (UNIFESP), São Paulo, São Paulo, Brazil.

[6] Department of Cardiovascular Surgery, Beneficência Portuguesa de São Paulo, São Paulo, São Paulo, Brazil.

[7] Department of Cardiovascular Surgery, Irmandade da Santa Casa de Misericórdia de São Paulo, São Paulo, São Paulo, Brazil.

[8] Department of Cardiovascular Surgery, Irmandade da Santa Casa de Misericórdia de Marília, Marília, São Paulo, Brazil.

[9] Departament of Cardiovascular Surgery, Universidade de São Paulo Hospital das Clínicas da Faculdade de Medicina de Ribeirão Preto, São Paulo, Brazil.

[10] Department of Cardiovascular Surgery, Irmandade da Santa Casa de Misericórdia de Piracicaba, Piracicaba, São Paulo, Brazil.

[11] Department of Cardiovascular Surgery, Hospital Paulo Sacramento, Jundiaí, São Paulo, Brazil.

***Corresponding author: E-mail: omar.mejia@incor.usp.br.

+these authors contributed equally to this work.

Supplementary file 2

Variables	Survivors patients N = 232		Avoidable death N= 58		*p* value
	N	%	N	%	
**Sex, male**	175	75.40%	36	62.10%	0.048*
**Age (years), mean, standard deviation**	58.40, ±20.25		61.46, ±9.54		0.049*
**Body mass index (kg/m^2), mean, standard deviation**	28.55, ±6.19		25.61, ±3.90		<0.001*
**Hematocrit (%), mean, standard deviation**	41.65, ±4.82		37.88, ±6.34		<0.001*
**Glycated hemoglobin, mean, standard deviation**	6.93, ±2.15		6.27, ±1.70		0.026*
**Creatinine (mg/dl) mean, standard deviation**	1.05, ±0.26		1.49, ±2.11		0.592
**Creatinine clearance (ml/min), mean, standard deviation**	84.84, ±25.34		67.66, ±25.86		<0.001*
**Left ventricular ejection fraction (%), mean, standard deviation**	59.78, ±8.67		59.74, ±10.14		0.855
**Pulmonary arterial pressure (mmHg), mean, standard deviation**	38.44, ±14.82		41.22, ±12.30		0.288
**New York Heart Association Functional class**					0.3
I	69	29.70%	13	22.40%	
II	92	39.70%	23	39.70%	
III	67	28.90%	19	32.80%	
IV	4	1.70%	3	5.20%	
**Recent acute myocardial infarction**	62	26.7	19	32.80%	0.413
**Rheumatic disease**	28	12.10%	9	15.50%	0.51
**Atrial fibrillation**	20	8.60%	4	6.90%	0.795
**Insulin-dependent diabetes mellitus**	15	6.50%	7	12.10%	0.166
**Stent**	19	8.20%	11	19%	0.027*
**Society of Thoracic Surgeons risk score, mean, standard deviation**	0.55, ±0.35		0.91, ±0.85		<0.001*
**EuroSCORE II, mean, standard deviation**	1.06, ±0.40		1.27, ±0.34		<0.001*
**Recalculated EuroSCORE II, mean, standard deviation**	-	-	2.34, ±1.96		-
**Status of the procedure**					0.918
Elective	162	69.80%	42	72.40%	
Urgent	68	29.30%	16	27.60%	
Emergency	2	0.90%	0	0%	
**Procedure**					0.004*
Isolated coronary artery bypass grafting	156	67.20%	29	50%	
Coronary artery bypass grafting + heart valve surgery	6	2.60%	6	10.30%	
Isolated aortic valve surgery	26	11.20%	8	13.80%	
Isolated mitral valve surgery	37	16%	8	13.80%	
Isolated tricuspid valve surgery	0	0%	1	1.70%	
≥ 2 combined valve surgery	6	2.60%	5	8.60%	
Others	1	0.40%	1	1.70%	

- not calculated.; * *p* value < 0,05.
