# Supplementary material for: Most deaths in low-risk cardiac surgery could be avoidable
Source: Sci Rep. 2021 Jan 13;11:1045. doi: 10.1038/s41598-020-80175-7 (PMC7806717; doi:10.1038/s41598-020-80175-7)
Supplement: Supplementary file 3 — Supplementary information 3. [file 41598_2020_80175_MOESM3_ESM.pdf]

Most deaths in low-risk cardiac surgery could be avoidable

Omar Asdrúbal Vilca Mejia[1,2,*], Gabrielle Barbosa Borgomoni[1], Eduardo Gomes Lima[1, +], Gustavo Pampolha Guerreiro[1,+], Luís Roberto Palma Dallan[1], Pedro Gabriel Melo de Barros e Silva[2,+], Marcelo Arruda Nakazone[3,+], Orlando Petrucci Junior[4,+], Walter José Gomes[5,+], Marco Antonio Praça de Oliveira[6,+], Alexandre Sousa[6,+], Valquíria Pelisser Campagnucci[7,+], Marcos Gradim Tiveron[8,+], Alfredo José Rodrigues[9,+] Rafael Ângelo Tineli[10,+], Roberto Rocha e Silva[11,+], Luiz Augusto Ferreira Lisboa[1], Fabio Biscegli Jatene[1].

[1] Department of Cardiovascular Surgery, Universidade de São Paulo Instituto do Coração (INCOR), São Paulo, São Paulo, Brazil.

[2] Department of Cardiovascular Surgery, Hospital Samaritano Paulista, São Paulo, São Paulo, Brazil.

[3] Department of Cardiovascular Surgery, Hospital De Base de São José do Rio Preto, São José de Rio Preto, São Paulo, Brazil.

[4] Department of Cardiovascular Surgery, Universidade Estadual de Campinas (UNICAMP), Campinas, São Paulo, Brazil.

[5] Department of Cardiovascular Surgery, Universidade Federal de São Paulo (UNIFESP), São Paulo, São Paulo, Brazil.

[6] Department of Cardiovascular Surgery, Beneficência Portuguesa de São Paulo, São Paulo, São Paulo, Brazil.

[7] Department of Cardiovascular Surgery, Irmandade da Santa Casa de Misericórdia de São Paulo, São Paulo, São Paulo, Brazil.

[8] Department of Cardiovascular Surgery, Irmandade da Santa Casa de Misericórdia de Marília, Marília, São Paulo, Brazil.

[9] Departament of Cardiovascular Surgery, Universidade de São Paulo Hospital das Clínicas da Faculdade de Medicina de Ribeirão Preto, São Paulo, Brazil.

[10] Department of Cardiovascular Surgery, Irmandade da Santa Casa de Misericórdia de Piracicaba, Piracicaba, São Paulo, Brazil.

[11] Department of Cardiovascular Surgery, Hospital Paulo Sacramento, Jundiaí, São Paulo, Brazil.

*****Corresponding author: E-mail: omar.mejia@incor.usp.br.

+these authors contributed equally to this work.

Supplementary file 3

Avoidable deaths

Patient	Surgical Procedure	Cardiac Death	Events leading up to death	Cause Of Death	POCMA Seminal Event	POCMA Category	Seminal Event in Hospitalization Phase
**1**	Coronary artery bypass grafting + mitral valve repair	Yes	Post-cardiac injury syndrome and low cardiac output	Cardiogenic shock	Risk > benefit	Judgment	Pre-operative phase
**2**	Coronary artery bypass grafting	No	Exploratory laparotomy by acute perforative abdomen	Septic shock	Sepsis prevention/treatment	Intensive care unit (keystone criteria)	Post-operative ICU phase (intensive care unit)
**3**	Coronary artery bypass grafting	Yes	Biventricular dysfunction	Cardiogenic shock	Myocardial Protection	Surgeon	Intra-operative phase
**4**	Coronary artery bypass grafting	Yes	Post-cardiac injury syndrome and low cardiac output	Cardiogenic shock	Medical status optimized	Patient preparation	Pre-operative phase
**5**	Mitral valve replacement	No	Prosthetic valve endocarditis, valve reoperation, acute renal syndrome	Acute endocarditis	Sepsis prevention/treatment	Intensive care unit (keystone criteria)	Post-operative ICU phase (intensive care unit)
**6**	Coronary artery bypass grafting	Yes	Reoperation for bleeding and low cardiac output	Cardiogenic shock	Technical	Surgeon	Intra-operative phase
**7**	Aortic valve replacement	Yes	Atrial fibrillation, ventricular fibrillation and cardiac arrest	Cardiogenic shock	-	Surveillance/recognition/Rx of decompensation	Post-operative floor phase (ward)
**8**	Mitral valve replacement + tricuspid valve repair	No	Pneumonia, acute renal failure	Septic shock	Sepsis prevention/treatment	Intensive care unit (keystone criteria)	Post-operative ICU phase (intensive care unit)
**9**	Mitral valve repair + tricuspid valve repair	Yes	Preoperative anemia and low cardiac output	Cardiogenic shock	Medical status optimized	Patient preparation	Pre-operative phase
**10**	Coronary artery bypass grafting	Yes	Preoperative anemia and low cardiac output	Cardiogenic shock	Medical status optimized	Patient preparation	Pre-operative phase
**11**	Coronary artery bypass grafting + mitral valve replacement	Yes	High glycated hemoglobin and low cardiac output	Cardiogenic shock	Medical status optimized	Patient preparation	Pre-operative phase
**12**	Coronary artery bypass grafting	Yes	Post-cardiac injury syndrome and low cardiac output	Cardiogenic shock	Risk > benefit	Judgment	Pre-operative phase
**13**	Coronary artery bypass grafting	Yes	Post-cardiac injury syndrome and low cardiac output	Cardiogenic shock	Risk > benefit	Judgment	Pre-operative phase
**14**	Coronary artery bypass grafting	Yes	Post-cardiac injury syndrome and low cardiac output	Cardiogenic shock	Risk > benefit	Judgment	Pre-operative phase
**15**	Coronary artery bypass grafting	Yes	Preoperative dialise, recent acute myocardial infarct, low cardiac output	Cardiogenic shock	Timing of surgery	Judgment	Pre-operative phase
**16**	Coronary artery bypass grafting	Yes	Ventricular fibrillation after closed chest, ST segment elevation and reoperation	Cardiogenic shock	Technical	Surgeon	Intra-operative phase
**17**	Coronary artery bypass grafting	Yes	Ventricular fibrillation in ICU, reoperation, low cardiac output	Cardiogenic shock	Technical	Surgeon	Intra-operative phase
**18**	Coronary artery bypass grafting	No	Massive bleeding, cardiorespiratory arrest, reoperation	Hypovolemic shock	Technical	Surgeon	Intra-operative phase
**18**	Coronary artery bypass grafting + aortic valve replacement + mitral valve replacement	Yes	Post-cardiac injury syndrome and low cardiac output	Cardiogenic shock	Risk > benefit	Judgment	Pre-operative phase
**20**	Tricuspid valve replacement + pulmonary valve replacement	Yes	Bleeding after surgery, acute renal failure, low cardiac output	Cardiogenic shock	Risk > benefit	Judgment	Pre-operative phase
**21**	Coronary artery bypass grafting	Yes	Preoperative abnormal coagulogram, reoperation for bleeding, low cardiac output	Cardiogenic shock	Risk > benefit	Judgment	Pre-operative phase
**22**	Aortic valve replacement	No	Pneumonia, acute renal failure, multiple organ failure	Multiple organ failure	-	Surveillance/recognition/Rx of decompensation	Post-operative ICU phase (intensive care unit)
**23**	Mitral valve repair	Yes	Persistence of significant mitral insufficiency, reoperation, bleeding, acute renal failure	Cardiogenic shock	Technical	Surgeon	Intra-operative phase
**24**	Aortic valve replacement	No	Higher preoperative risk, aortic valve endocarditis, multiple organ failure	Septic shock	Risk > benefit	Judgment	Pre-operative phase
**25**	Aortic valve repair + mitral valve replacement + tricuspid valve repair	No	Higher preoperative risk, severe postoperative clinical decompensation, acute renal failure, sepsis	Septic shock	Medical status optimized	Patient preparation	Pre-operative phase
**26**	Mitral valve replacement	No	Massive bleeding, reoperation for bleeding, acute renal failure, sepsis	Septic shock	Technical	Surgeon	Intra-operative phase
**27**	Mitral valve replacement	Yes	Ventricular dysfunction after surgery, low cardiac output	Cardiogenic shock	Myocardial protection	Surgeon	Intra-operative phase
**28**	Coronary artery bypass grafting	Yes	Severe hemodynamic instability, low cardiac output	Cardiogenic shock	Risk identification	Patient evaluation	Pre-operative phase
**29**	Coronary artery bypass grafting + aortic valve replacement	Yes	Post-cardiac injury syndrome, low cardiac output	Cardiogenic shock	Risk identification	Patient evaluation	Pre-operative phase
**30**	Coronary artery bypass grafting	Yes	Unidentified abdominal aortic aneurysm, severe hemodynamic instability and low cardiac output	Cardiogenic shock	Risk identification	Patient evaluation	Pre-operative phase
**31**	Coronary artery bypass grafting	Yes	Off pump surgery, laceration of the right ventricle, hemodynamic instability, low cardiac output	Cardiogenic shock	Judgment	Surgeon	Intra-operative phase
**32**	Aortic valve replacement	Yes	Minitoracotomy, postoperative bleeding, reoperation, hemodynamic instability	Cardiogenic shock	Judgment	Surgeon	Intra-operative phase
**33**	Coronary artery bypass grafting	Yes	Prolonged cardiopulmonary bypass time, hemodynamic instability, ventricular dysfunction	Cardiogenic shock	Judgment	Surgeon	Intra-operative phase
**34**	Mitral valve replacement	No	Pneumonia, sepsis, hemodynamic instability	Septic shock	Sepsis prevention/treatment	Intensive care unit (keystone criteria)	Post-operative ICU phase (intensive care unit)
**35**	Coronary artery bypass grafting	Yes	Reoperation for bleeding at the aorta suture, hemodynamic instability, low cardiac output	Cardiogenic shock	Technical	Surgeon	Intra-operative phase
**36**	Coronary artery bypass grafting	No	Surgical wound infection, sepsis, hemodynamic instability	Septic shock	-	Surveillance/recognition/Rx of decompensation	Post-operative ICU phase (intensive care unit)
**37**	Coronary artery bypass grafting	No	History of liver transplantation, reoperation for bleeding, acute renal failure, hemodynamic instability	Multiple organ failure	Risk identification	Patient evaluation	Pre-operative phase
**38**	Coronary artery bypass grafting	Yes	Reoperation for bleeding, acute renal failure, hemodynamic instability	Cardiogenic shock	Technical	Surgeon	Intra-operative phase
**39**	Mitral valve replacement + tricuspid valve repair	Yes	Higher preoperative risk, hemodynamic instability, low cardiac output	Cardiogenic shock	Risk identification	Patient evaluation	Pre-operative phase
**40**	Aortic valve replacement	No	Prolonged cardiopulmonary bypass time, reoperation for bleeding, hemodynamic instability	Vasoplegic shock	Technical	Surgeon	Intra-operative phase
**41**	Coronary artery bypass grafting	Yes	Reoperation for bleeding, hemodynamic instability, low cardiac output	Cardiogenic shock	Technical	Surgeon	Intra-operative phase
**42**	Mitral valve replacement + tricuspid valve repair	Yes	Higher preoperative risk, hemodynamic instability, low cardiac output	Cardiogenic shock	Risk identification	Patient evaluation	Pre-operative phase
**43**	Aortic valve replacement	Yes	Reoperation for bleeding, hemodynamic instability, low cardiac output	Cardiogenic shock	Technical	Surgeon	Intra-operative phase
**44**	Coronary artery bypass grafting + aortic valve replacement	No	Severe postoperative clinical decompensation, sepsis	Septic shock	Sepsis prevention/treatment	Intensive care unit (keystone criteria)	Post-operative ICU phase (intensive care unit)
**45**	Aortic valve replacement	No	Pneumonia, sepsis, hemodynamic instability	Septic shock	Sepsis prevention/treatment	Intensive care unit (keystone criteria)	Post-operative ICU phase (intensive care unit)
**46**	Aortic valve replacement	No	Acute endocarditis, sepsis, hemodynamic instability	Septic shock	-	Sepsis prevention/treatment	Post-operative floor phase (ward)
**47**	Coronary artery bypass grafting	Yes	Higher preoperative risk, hemodynamic instability, low cardiac output	Cardiogenic shock	Risk identification	Patient evaluation	Pre-operative phase
**48**	Coronary artery bypass grafting	Yes	Acute myocardial infarction, ventricular assist device, low cardiac output	Cardiogenic shock	Judgment	Surgeon	Intra-operative phase
**49**	Mitral valve replacement	Yes	Reoperation for bleeding, hemodynamic instability, low cardiac output	Cardiogenic shock	Technical	Surgeon	Intra-operative phase
**50**	Coronary artery bypass grafting	No	Severe postoperative clinical decompensation, sepsis	Septic shock	Sepsis prevention/treatment	Intensive care unit (keystone criteria)	Post-operative ICU phase (intensive care unit)
**51**	Coronary artery bypass grafting	Yes	Reoperation for bleeding, cardiac arrhythmia, hemodynamic instability	Cardiogenic shock	Technical	Surgeon	Intra-operative phase
**52**	Mitral valve replacement	Yes	Mitral valve replacement after attempted repair, reoperation due to bleeding, low cardiac output	Cardiogenic shock	Judgment	Surgeon	Intra-operative phase
**53**	Coronary artery bypass grafting	Yes	Higher preoperative risk, Hemodynamic instability, low cardiac output	Cardiogenic shock	Risk identification	Patient evaluation	Pre-operative phase
**54**	Coronary artery bypass grafting	No	Surgical wound infection, severe postoperative clinical decompensation, sepsis	Septic shock	-	Surveillance/recognition/Rx of decompensation	Post-operative floor phase (ward)
**55**	Coronary artery bypass grafting	Yes	Acute myocardial infarction, ventricular assist device, ventricular arrhythmia, low cardiac output	Cardiogenic shock	Judgment	Surgeon	Intra-operative phase
**56**	Coronary artery bypass grafting	No	Severe postoperative clinical decompensation, sepsis	Septic shock	-	Surveillance/recognition/Rx of decompensation	Post-operative ICU phase (intensive care unit)
**57**	Mitral valve replacement	No	Surgical wound infection, severe postoperative clinical decompensation, sepsis	Septic shock	Sepsis prevention/treatment	Intensive care unit (keystone criteria)	Post-operative ICU phase (intensive care unit)
**58**	Coronary artery bypass grafting	No	Prolonged hospital stay, mediastinitis, sepsis	Septic shock	Sepsis prevention/treatment	Intensive care unit (keystone criteria)	Post-operative ICU phase (intensive care unit)
