# Supplementary material for: Most deaths in low-risk cardiac surgery could be avoidable
Source: Sci Rep. 2021 Jan 13;11:1045. doi: 10.1038/s41598-020-80175-7 (PMC7806717; doi:10.1038/s41598-020-80175-7)
Supplement: Supplementary file 4 — Supplementary information 4. [file 41598_2020_80175_MOESM4_ESM.pdf]

Most deaths in low-risk cardiac surgery could be avoidable

Omar Asdrúbal Vilca Mejia[1,2,*], Gabrielle Barbosa Borgomoni[1], Eduardo Gomes Lima[1, +], Gustavo Pampolha Guerreiro[1,+], Luís Roberto Palma Dallan[1], Pedro Gabriel Melo de Barros e Silva[2,+], Marcelo Arruda Nakazone[3,+], Orlando Petrucci Junior[4,+], Walter José Gomes[5,+], Marco Antonio Praça de Oliveira[6,+], Alexandre Sousa[6,+], Valquíria Pelisser Campagnucci[7,+], Marcos Gradim Tiveron[8,+], Alfredo José Rodrigues[9,+] Rafael Ângelo Tineli[10,+], Roberto Rocha e Silva[11,+], Luiz Augusto Ferreira Lisboa[1], Fabio Biscegli Jatene[1].

[1] Department of Cardiovascular Surgery, Universidade de São Paulo Instituto do Coração (INCOR), São Paulo, São Paulo, Brazil.

[2] Department of Cardiovascular Surgery, Hospital Samaritano Paulista, São Paulo, São Paulo, Brazil.

[3] Department of Cardiovascular Surgery, Hospital De Base de São José do Rio Preto, São José de Rio Preto, São Paulo, Brazil.

[4] Department of Cardiovascular Surgery, Universidade Estadual de Campinas (UNICAMP), Campinas, São Paulo, Brazil.

[5] Department of Cardiovascular Surgery, Universidade Federal de São Paulo (UNIFESP), São Paulo, São Paulo, Brazil.

[6] Department of Cardiovascular Surgery, Beneficência Portuguesa de São Paulo, São Paulo, São Paulo, Brazil.

[7] Department of Cardiovascular Surgery, Irmandade da Santa Casa de Misericórdia de São Paulo, São Paulo, São Paulo, Brazil.

[8] Department of Cardiovascular Surgery, Irmandade da Santa Casa de Misericórdia de Marília, Marília, São Paulo, Brazil.

[9] Departament of Cardiovascular Surgery, Universidade de São Paulo Hospital das Clínicas da Faculdade de Medicina de Ribeirão Preto, São Paulo, Brazil.

[10] Department of Cardiovascular Surgery, Irmandade da Santa Casa de Misericórdia de Piracicaba, Piracicaba, São Paulo, Brazil.

[11] Department of Cardiovascular Surgery, Hospital Paulo Sacramento, Jundiaí, São Paulo, Brazil.

*Corresponding author: E-mail: omar.mejia@incor.usp.br.

+these authors contributed equally to this work.

Supplementary file 4

Unavoidable deaths

Patient	Surgical procedure	Events leading up to death	POCMA category	Seminal Event in Hospitalization phase
1	Coronary artery bypass grafting + aortic valve replacement	Drug Encephalopathy by Cefepime drug	Catastrophic event	Intra-operative phase
2	Aortic valve replacement	Severe and Refractory Systemic inflammatory response syndrome (SIRS) without identifiable risk factors	Catastrophic event	Post-operative ICU phase (intensive care Unit)
3	Mitral valve repair	Important commitment of cerebral self-regulation and Narcosis after surgery	Catastrophic event	Post-operative ICU phase (intensive care Unit)
4	Mitral valve replacement	Severe and Refractory Pulmonary Hipertension without identifiable risk factors. Normal cardiac function.	Catastrophic event	Intra-operative phase
5	Aortic valve replacement	Patient with normal postoperative echocardiogram evolves with cardiac arrest with pulseless electrical activity.	Catastrophic event	Post-operative floor phase (Ward)
6	Coronary artery bypass grafting	Patient evolved with perforation of the abdominal aorta after passage of an intraortic balloon	Catastrophic event	Intra-operative phase
7	Coronary artery bypass grafting	Severe and Refractory Acute Respiratory Insufficiency without identifiable risk factors	Catastrophic event	Post-operative ICU phase (intensive care Unit)
8	Coronary artery bypass grafting	Severe and Refractory Vasoplegic Syndrome without identifiable risk factors	Catastrophic event	Post-operative ICU phase (intensive care Unit)
9	Mitral valve replacement	Severe and Refractory Arrhythmias without identifiable risk factors. Cardiac catheterization and echocardiography smoothly	Catastrophic event	Post-operative ICU phase (intensive care Unit)
10	Coronary artery bypass grafting	Severe and Refractory Arrhythmias without identifiable risk factors	Catastrophic event	Post-operative ICU phase (intensive care Unit)
11	Aortic valve replacement	Sepsis without response to antibiotic treatment. Prevention protocols were applied	Catastrophic event	Post-operative ICU phase (intensive care Unit)
12	Coronary artery bypass grafting	Surgery with incomplete revascularization due to disease in the ascending aorta. During left main coronary artery angioplasty evolves with ventricular fibrillation and cardiac arrest	Catastrophic event	Post-operative floor phase (Ward)
13	Aortic valve replacement	Severe and Refractory Status Epilepticus and Multiple organ dysfunction syndrome	Catastrophic event	Post-operative ICU phase (intensive care Unit)
14	Mitral valve replacement	Severe and Refractory Vasoplegic Syndrome without identifiable risk factors	Catastrophic event	Post-operative ICU phase (intensive care Unit)
15	Coronary artery bypass grafting	Acute mesenteric ischemia without identifiable risk factors	Catastrophic event	Post-operative ICU phase (intensive care Unit)
16	Coronary artery bypass grafting + aortic valve replacement	Iatrogenic Aortic Dissection at the end of surgery	Catastrophic event	Intra-operative phase
17	Coronary artery bypass grafting	Severe and Refractory Vasoplegic Syndrome without identifiable risk factors	Catastrophic event	Post-operative ICU phase (intensive care Unit)
18	Coronary artery bypass grafting	Severe and Refractory Bleeding by Cogulopathy without identifiable risk factors	Catastrophic event	Intra-operative phase
19	Coronary artery bypass grafting	Severe and Refractory Postoperative clinical decompensation without identifiable risk factors	Catastrophic event	Post-operative ICU phase (intensive care Unit)
